# Early prediction of prostate cancer risk in younger men using polygenic risk scores and electronic health records

**DOI:** 10.1002/cam4.4934

**Published:** 2022-06-25

**Authors:** Amita Varma, Jenish Maharjan, Anurag Garikipati, Myrna Hurtado, Sepideh Shokouhi, Qingqing Mao

**Affiliations:** ^1^ Dascena Inc. Houston Texas USA; ^2^ Montera Inc. San Francisco CA USA

**Keywords:** algorithms, decision support, machine learning, polygenic risk score, prostate cancer

## Abstract

**Background:**

Prostate cancer (PCa) screening is not routinely conducted in men aged 55 and younger, although this age group accounts for more than 10% of cases. Polygenic risk scores (PRSs) and patient data applied toward early prediction of PCa may lead to earlier interventions and increased survival. We have developed machine learning (ML) models to predict PCa risk in men 55 and under using PRSs combined with patient data.

**Methods:**

We conducted a retrospective study on 91,106 male patients aged 35–55 using the UK Biobank database. Five gradient boosting models were developed and validated utilizing routine screening data, PRSs, additional clinical data, or combinations of the three.

**Results:**

Combinations of PRSs and patient data outperformed models that utilized PRS or patient data only, and the highest performing models achieved an area under the receiver operating characteristic curve of 0.788. Our models demonstrated a substantially lower false positive rate (35.4%) in comparison to standard screening using prostate‐specific antigen (60%–67%).

**Conclusion:**

This study provides the first preliminary evidence for the use of PRSs with patient data in a ML algorithm for PCa risk prediction in men aged 55 and under for whom screening is not standard practice.

## Graphical abstract

Prostate cancer screening is not routinely conducted in men aged 55 and younger, despite the fact that more than 10% of cases are in this age group. We developed machine learning models to predict prostate cancer risk in men 55 and under using polygenic risk scores and other patient data.

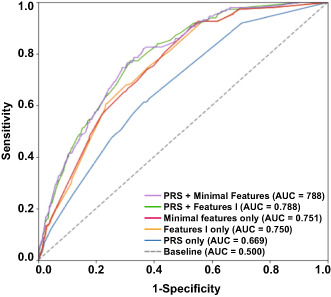

## 1 INTRODUCTION

Prostate cancer (PCa) is the second most common cancer in men and responsible for 375,000 deaths worldwide.^1^ Although it presents an indolent clinical course, PCa still remains a major health burden with mortality rates expected to rise 1.05% by 2040.^2^ PCa is generally asymptomatic in the early and later stages.^3^, ^4^ Routine cancer screening can prevent future health complications by facilitating early detection and allowing for timely intervention. The most common screening methods for PCa are the digital rectal examination (DRE) and prostate‐specific antigen (PSA) test. The largest conducted trial of DRE and PSA screening demonstrated the usefulness of screening with a subsequent risk reduction in PCa‐related deaths of up to 49%.^5^ However, there is controversy surrounding the effectiveness of PSA screening as false positive results, overdiagnosis and overtreatment are associated with the use of this screening tool.^6^ In 2012, the United States Preventive Services Task Force issued a recommendation discouraging routine PCa screening in men regardless of risk factors, causing high‐grade cases to increase by 11.3%.^4^ Further efforts are warranted to improve current PCa initial screening approaches and methods.

Screening is generally recommended for men aged 55 and older, as the majority of PCa cases are diagnosed in older men. Although the average age of PCa diagnosis is 66, with the highest incidence seen in those older than 65,^7^ more than 10% of cases occur in men aged 55 and younger^8^ and current research indicates that younger men diagnosed with high‐grade PCa have an overall poorer prognosis.^9^ Developing an accurate screening tool to predict the risk of PCa for patients younger than the standard screening age would therefore allow for earlier identification of those younger patients at risk and potentially reduce the public health burden.

The high heritability of PCa^10^ demonstrates that genetic factors play a considerable role in its development. Several genome‐wide association studies have identified over 200 single nucleotide polymorphisms (SNPs) that are associated with an increased risk of PCa.^11^, ^12^, ^13^ These genetic variants can be combined to determine an individual's polygenic risk score (PRS), and PRSs have been demonstrated to have a large clinical utility potential for numerous diseases, including PCa.^14^

Prostate cancer is also associated with additional known risk factors, such as age and ethnicity,^15^ that can be routinely entered into electronic health records. PRSs along with patient data may be used for earlier and more accurate predictions of PCa, leading to earlier interventions, increased survival, and reduced healthcare costs. We have developed and validated machine learning (ML) models to predict PCa diagnosis specifically in younger men (age ≤ 55) based on PRS and relevant patient data. This risk assessment screening method is not contingent on the use of PSA or DRE results.

## 2 METHODS

### 2.1 Data source

Data from 502,460 participants in the UK Biobank (UKBB) were analyzed in this retrospective study. UKBB is a longitudinal electronic health record repository that incorporates clinical and genetic data. Patient data from hospitals and UKBB assessment centers between January 2007 and June 2020 were used in this study. Prior to use, passive patient data gathering and de‐identification were conducted in compliance with the Health Insurance Portability and Accountability Act. The use of de‐identified retrospective data is classified as a non‐human subject study and exempt from Institutional Review Board approval.

### 2.2 Cohort definition and gold standard

We included all male UKBB participants aged 35–55 who had genotypic data. The gold standard labels for a positive PCa diagnosis were defined using data from two fields: International Classification of Diseases, Tenth Revision, Clinical Modification (ICD‐10) diagnoses, and self‐reported cancer code. The ICD‐10 code used to define PCa was C61—malignant neoplasm of prostate, and the self‐reported cancer code was 1044—prostate cancer. Any patient fitting either of these two criteria were labeled as positive. Those that had a PCa diagnosis prior to this visit were excluded. All other patients were considered negative cases.

### 2.3 Genetic data and PRS

The PRSs were created using the PRSice tool (https://www.prsice.info/quick_start/).^16^ The polygenic score (PGS) weights found on the PGS Catalog website (https://www.pgscatalog.org/score/PGS000333/)^17^ were used to generate the PRSs for every participant. The genome‐wide association study for this set of weights was performed on a cohort of European ancestry for PCa among other traits, and included more than 170 validated SNPs associated with PCa.^11^ This set of weights were then trained and validated on the FINRISK biobank cohort.^17^ The study reported weights and summary statistics of 6,606,785 SNPs. The UKBB cohort had a total of 784,256 variants out of which 541,268 variants overlapped with the variants reported by the study, and included more than 200 validated PCa SNPs.^12^

### 2.4 Machine learning algorithm, input features, and prediction models

We used XGBoost, a gradient boosting algorithm,^18^ implemented in Python. This algorithm was chosen because it allows the analysis of contributions of individual features to the algorithm results. Five models were developed: (1) PRSs only, (2) Features I: utilizing only age, father's history, sibling history, and ethnicity, (3) PRSs + Features I, (4) Minimal features: age, father's history, and body mass index (BMI), and (5) PRSs + minimal features. Four additional models were investigated: (1) Features II: Features I + BMI, smoking status, glycated hemoglobin, C‐reactive protein, and insulin‐like growth factor 1, (2) PRS + Features II, (3) Features III: Features II + number of sex partners, diabetes diagnosis, and diabetes medication and (4) PRS + Features III. The selection of these additional variables is based on previous UKBB studies that developed PCa prediction models and identified modified risk due to smoking status,^19^ glycated hemoglobin,^20^ C‐reactive protein,^20^ insulin‐like growth factor,^20^ number of sex partners,^19^ diabetes diagnosis,^21^ and diabetes medication^21^ as important risk factors and predictors of PCa. We partitioned the dataset into training (60%), validation (20%), and hold‐out testing (20%) splits prior to training of the model. The validation set was used during training to validate the model performance. The hold‐out test set was not seen by the model during the training or validation phase. Results are reported for the hold‐out test set. The model was trained to predict PCa up to 11 years, the maximum time between the patient's visit to the health facility used for training and the first diagnosis of PCa in the data set. Missing values in the continuous features were filled as null, and missing values in categorical features were filled with the appropriate data code for “Unknown” or “No response”. Family history features are binary features, created by checking the presence of the code for PCa in the appropriate columns for father and sibling history. The hyperparameters of the model were tuned on the validation set using a threefold grid search cross‐validation approach. The hyperparameters that were tuned were *eta* (learning rate), *gamma* (minimum loss reduction to split), and *lambda* (L2 regularization term). The number of estimators were fixed to be 100, and the maximum depth to be 6.

### 2.5 Statistical analysis

The performance of each model was evaluated on the 20% hold‐out test set with respect to the area under the receiver operating characteristic (AUROC), sensitivity, specificity, diagnostic odds ratio, and positive and negative likelihood ratios. The threshold for predicting labels was calculated by setting the minimum sensitivity value to 0.800. 95% confidence intervals for these metrics were constructed using 1000 bootstrapped samples. We conducted a SHapely Additive ExPlanations (SHAP) plot^22^ to evaluate feature importance.

## 3 RESULTS

### 3.1 Subject characteristics

**FIGURE 1:**
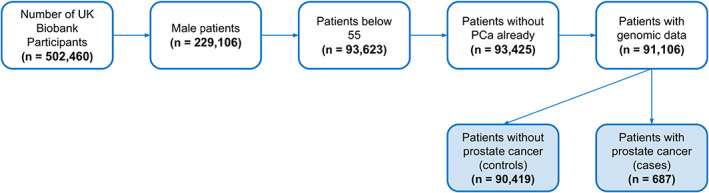
Attrition chart for inclusion criteria of UK Biobank participants. PCa, Prostate cancer.

There were 502,460 UKBB participants before exclusion of any patients. The number of patients were 229,106 after excluding female patients. After exclusion of male patients over the age of 55 and those with a prior PCa diagnosis, a total of 91,106 men were included in the study: 90,419 control participants and 687 participants with a PCa diagnosis. Figure 1 represents the attrition chart. Table 1 summarizes the patient characteristics for positive cases (participants with PCa diagnosis) and controls (participants with no PCa diagnosis). Group differences were calculated with Fisher's exact test. Age (*p* < 0.0001) and black ethnicity (*p* < 0.0001), showed significant group differences. Table 2 provides the input features for the five ML models: PRS only, Features I (age, father's history, sibling history, and ethnicity), PRS + Features I, Minimal Features (age, father's history, and BMI), PRS + Minimal Features. Table S1 lists the input variables for the four additional ML models: Features II, PRS+ Features II, Features III, and PRS + Features III.

**TABLE 1:** Demographic data and other patient characteristics for individuals with and without prostate cancer (PCa) included in the analysis

Demographics	With PCa (*n* = 687)	Without PCa (*n* = 90,419)	*p*‐value
Age			
35–45	29 (4.2%)	23,334 (25.8%)	<0.0001
45–55	658 (95.8%)	67,085 (74.2%)	<0.0001
Ethnicity			
White	624 (90.8%)	82,384 (91.1%)	0.978
Black	37 (5.4%)	2239 (2.5%)	<0.0001
Asian	13 (1.9%)	3377 (3.7%)	0.010
Mixed	7 (1.0%)	683 (0.8%)	0.375
Other	6 (0.9%)	1736 (1.9%)	0.048
BMI			
Mean	27.53	27.76	0.186
Range (Min–Max)	16.75–43.16	14.87–63.44	–
Smoking status			
Current smoker	95 (13.8%)	13,886 (15.4%)	0.369
Previous smoker	185 (26.9%)	24,721 (27.3%)	0.901
Never smoker	406 (59.1%)	51,413 (56.9%)	0.549
Did not answer	1 (0.1%)	399 (0.4%)	0.381

Abbreviations: BMI, body mass index.

**TABLE 2:** Input variables for the five machine learning models: PRS only, Features I, PRS + Features I, Minimal Features, and PRS + Minimal Features

	Machine learning model				
Input variables	(1) PRS only	(2) Features I	(3) PRS + Features I	(4) Minimal Features	(5) PRS + Minimal Features
Genetic	PRS		PRS		PRS
Demographics		Age	Age	Age	Age
		Father's history	Father's history	Father's history	Father's history
		Sibling history	Sibling history		
		Ethnicity	Ethnicity		
Clinical measurements				BMI	BMI

Abbreviations: BMI, body mass index; PRS, polygenic risk score.

### 3.2 ML algorithm performance

**FIGURE 2:**
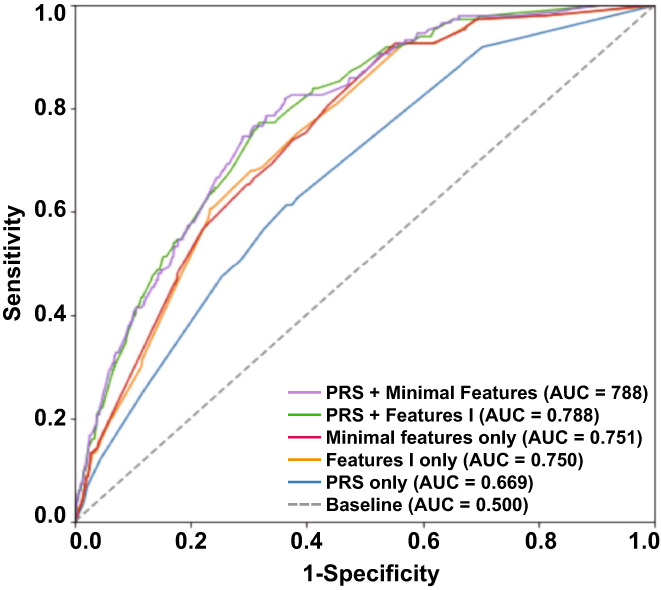
Receiver operating characteristic (ROC) curves of the five machine learning algorithm models for risk prediction of prostate cancer (PCa): PRSs only, Features I, PRSs + Features I, Minimal Features, and PRSs + Minimal Features. AUC, area under the curve; PRS, polygenic risk score.

Figure 2 shows the ROC curves for the five models. Table 3 summarizes the performance metrics of all five models and includes the AUROC, sensitivity, specificity, diagnostic odds ratio, false positive rates, and positive and negative likelihood ratios. The PRS + Features I and PRS + Minimal Features models' performance were comparable and demonstrated the highest AUROCs, 0.788 (95% CI = 0.758–0.819) and 0.788 (95% CI = 0.757–0.820), respectively. At a sensitivity of 0.800, the PRS + Features I model demonstrated a specificity of 0.629, and the PRS + Minimal Features model a specificity of 0.646. The PRS + Minimal Features model had a false positive rate of 35.4%. The performance metrics of the four additional models (Features II, PRS + Features II, Features III, and PRS + Features III) are presented in Table S2.

**TABLE 3:** Performance metrics of all five machine learning algorithm models

	Only PRS scores	Features I only	PRS + Features I	Minimal Features only	PRS + Minimal Features
AUROC	0.669 (0.634, 0.708)	0.750 (0.714, 0.781)	0.788 (0.758, 0.819)	0.751 (0.714, 0.784)	0.788 (0.757, 0.820)
Sensitivity	0.920 (0.877, 0.963)	0.807 (0.743, 0.870)	0.800 (0.736, 0.864)	0.807 (0.743, 0.870)	0.800 (0.736, 0.864)
Specificity	0.295 (0.289, 0.302)	0.552 (0.545, 0.560)	0.629 (0.622, 0.636)	0.563 (0.556, 0.570)	0.646 (0.639, 0.653)
DOR	4.819 (4.229, 5.410)	5.151 (4.744, 5.557)	6.783 (6.382, 7.184)	5.377 (4.971, 5.784)	7.299 (6.897, 7.700)
LR+	1.306 (1.244, 1.370)	1.802 (1.664, 1.953)	2.157 (1.986, 2.341)	1.846 (1.704, 2.000)	2.260 (2.081, 2.454)
LR‐	0.271 (0.157, 0.466)	0.350 (0.252, 0.485)	0.318 (0.231, 0.438)	0.343 (0.248, 0.476)	0.310 (0.225, 0.426)
FPR	0.705 (0.698, 0.711)	0.448 (0.440, 0.455)	0.371 (0.364, 0.378)	0.437 (0.430, 0.444)	0.354 (0.347, 0.361)

Abbreviations: AUROC, area under the receiver operating characteristic; DOR, diagnostic odds ratio; FPR, false positive rate; LR+, likelihood ratio positive; LR−, likelihood ratio negative; PRS, polygenic risk score.

### 3.3 Feature importance

**FIGURE 3:**
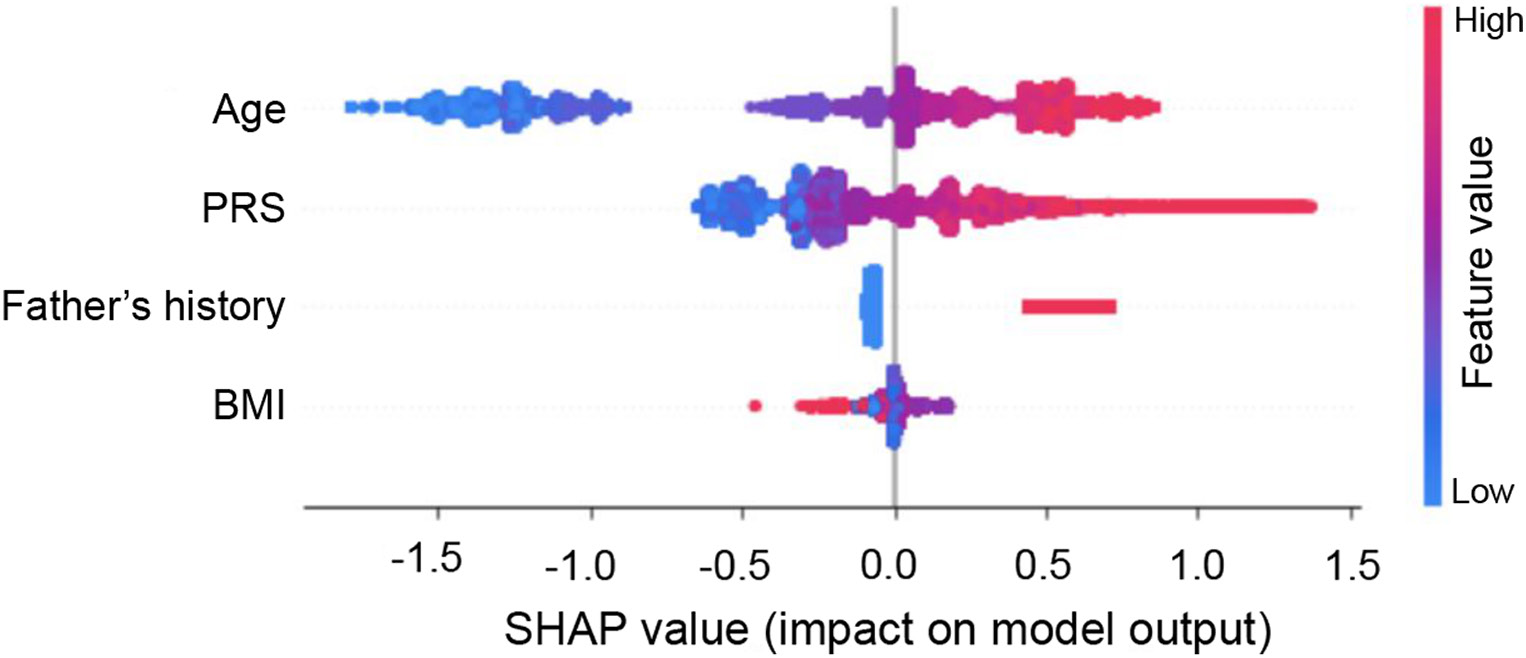
SHapely Additive exPlanations (SHAP) plot of the PRS + Minimal Features model. Display of the top predictor correlations and distribution of feature importance. PRS, polygenic risk score; BMI, body mass index.

The SHAP plot (Figure 3) shows the features with the highest contribution to the XGB results for the PRS + Minimal Features model. Age, PRS, and father's PCa history were identified as the top features having a positive association with PCa risk, whereas higher BMI was associated with lower risk. Sibling history and ethnicity were also identified as high‐importance predictors in the Features I model; however, inclusion of PRS resulted in a sharp decrease in their feature importance. Figure S1 presents the SHAP plots of the Features I, PRSs + Features I, Features II, PRS + Features II model, and Minimal Features ML models. While the risk factors featured in our additional models are not typically used in routine clinical examinations for PCa, investigation in previously published ML‐based studies prompted analysis in our study. Apart from glycated hemoglobin, the supplementary features did not improve model performance and thus are not the main features of focus in our study.

## 4 DISCUSSION

### 4.1 Summary of the study

This is the first study demonstrating the utility of ML algorithms for PCa risk assessment in younger men who have not reached the recommended age for routine PCa screening. We achieved the same accuracy as the PRS + Features I model with fewer inputs (PRS + Minimal Features) and successfully created a risk assessment tool for identifying high‐risk individuals among men aged 55 and younger. PCa incidence in men of this age group has been steadily increasing over the last few decades and is expected to continue rising. Younger men with high grade PCa have a significantly diminished overall survival and disease‐specific survival compared to older men.^23^, ^24^ Our ML‐based prediction model may aid in the early detection of PCa in young at‐risk individuals to prompt further examination and provide an opportunity for early treatment and prevention options.

### 4.2 Significance and impact of PRS in PCa prediction models

There are notable biological differences between early and late onset PCa, which can have significant clinical implications.^25^ The early onset of PCa in younger men is thought to be largely attributed to genetic factors.^26^ This is consistent with the presence of PRS and paternal PCa history among the top features of the ML prediction models. Although familial history is a known PCa risk factor, using genetic data in the form of a PRS can provide a more objective risk profile that is not contingent upon accurate information from an individual's family members.^27^ Previous studies have shown the clinical utility of using PRS data by accurately predicting PCa risk and demonstrating that this method may also aid in reducing overdiagnosis.^28^, ^29^ As research continues to identify new PCa susceptibility loci,^11^ we expect that future ML models will incorporate improved PRSs in parallel with new genetic discoveries.

### 4.3 Importance of other features

When PRS was not included in our model, ethnicity was among the significant features. PCa is known to be disproportionately higher in Black men,^30^, ^31^ which is consistent with the significant overrepresentation of Black men with PCa cases in comparison to other racial groups in our study. Sibling history was an additional model feature determined to be of high importance, although the addition of PRS to the ML model substantially reduced its importance as a predictor. Incorporation of PRS diminished the need for these additional features and maintained high accuracy in our Minimal Features model. Interaction and overlapping effects between PRS and race/ethnicity, sibling history and glycated hemoglobin were not explored in this study and warrant further investigation.

Age was identified as the most important feature in predicting PCa, in agreement with established literature.^7^ Addition of other clinical data (BMI) also slightly improved performance metrics. BMI has been reported to influence PCa aggressiveness, however, the mechanisms are not yet known.^32^, ^33^, ^34^ We observed a negative association between BMI and PCa risk. Similar findings were reported by Giovannuci et al.^35^ who found a lower risk of PCa in men with higher BMI only if they were younger (<60 years old) or had a family history of PCa, and attributed their findings to the complex relationship between obesity and various hormones.

### 4.4 Comparison with other models

Previous studies in older men (ranging from 55 to 80 years) have reported that the inclusion of PRS data improves the performance of different PCa prediction models.^36^, ^37^, ^38^, ^39^ Oh et al.^38^ identified several ethnicity‐specific SNPs with moderate predictive performance (an AUROC of 0.637) in men 60 years and older. Aly et al.^39^ developed a baseline model in men 80 years and under based on age, PSA levels and familial history and determined that the inclusion of PRS improved performance metrics (AUROC of 0.64–0.67) as well as reduced the number of required diagnostic biopsies. Our models did not rely on the use of PSA tests, which can be associated with false positive results leading to biopsy complications,^40^ particularly in younger men who have a distinctive phenotype characterized by early‐onset PCa where poorly differentiated adenocarcinoma may impact the accuracy of PSA as a PCa risk predictor.^9^ The distribution of baseline PSA levels in younger men has also been reported to vary across ethnic groups,^41^ suggesting that screening guidelines involving PSA values in younger men may not be applicable to all population groups. Our model demonstrated a substantial reduction in false positive rates for ML PCa screening compared to using PSA serum tests: 35.4% for our PRS + Minimal Features model in men aged 35–55, versus 60% to 67% for PSA screening in men aged 55–71.^42^ This was also a considerable improvement from the 70.5% false positive rate of our PRS only model, which is comparable to current false positive rates of PSA screening in older men. Our ML models' combination use of genetic and patient data demonstrated increased accuracy in the identification of PCa risk in a younger cohort of men.

### 4.5 Study limitations

There are several limitations to this study. First, this work was conducted retrospectively, therefore we cannot determine how this model would perform in prospective clinical practice. Additionally, PRS studies that use cohorts of mainly European descent, as is the case with our dataset, may not be generalizable to other populations and may affect risk prediction accuracy for individuals of non‐European ancestry.^43^ Information on Gleason grades was not available in our database. Our presented model aims to prompt evaluation and thorough testing in patients at high‐risk for PCa. Consideration of Gleason grades and genetic variants is important for management and treatment, and are an area for future research. Other PCa risk assessment tools^44^, ^45^ require PSA or DRE measures, which were not available for direct comparison of risk assessment in this population. The clinical applicability and statistical power of these findings are limited by a relatively small number of cases, particularly among the population groups with the youngest ages and nonwhite race categories, as well as a limited number of race‐agnostic SNPs. Adjustments for PRSs were not made using race‐specific SNPs; race should be accounted for (or considered) in calculating PRS in future studies. This work provides promising preliminary evidence for PCa risk evaluation in younger men and warrants future studies that should include validation of our ML algorithm in a prospective clinical setting and assessing how patient care and outcomes are affected.

## 5 CONCLUSION

Machine learning algorithms which include PRS information and basic patient data can provide risk assessment for PCa in a young population not routinely screened. Efforts to identify men at risk in earlier age groups can help decrease the burden of PCa. Future work to support implementation of ML algorithms for PCa risk assessment of younger men in clinical practice is needed.

## AUTHOR CONTRIBUTIONS

Amita Varma: Formal analysis, software, visualization, and writing—original draft. Jenish Maharjan: Formal analysis and software. Myrna Hurtado: writing—original draft and writing—review and editing. Sepideh Shokouhi: writing—original draft and writing—review and editing. Anurag Garikipati: Conceptualization. Qingqing Mao: Conceptualization.

## FUNDING INFORMATION

The authors received no public funding for this work.

## CONFLICT OF INTERESTS

The authors are or have been employees or contractors of Dascena, Inc.

## ETHICS APPROVAL AND CONSENT TO PARTICIPATE

Data were collected passively and de‐identified in compliance with the Health Insurance Portability and Accountability Act, thus, this study was considered non‐human subjects research and did not require Institutional Review Board approval.

## Supporting information


**Appendix S1** Supporting Information

## Data Availability

The data used in this study are available from UKBB and may be accessed by completing an application via https://www.ukbiobank.ac.uk/register‐apply/. The ML algorithm code developed in this study is proprietary and not publicly available.
