# Supplementary material for: Early prediction of prostate cancer risk in younger men using polygenic risk scores and electronic health records
Source: Cancer Med. 2022 Jun 25;12(1):379–86. doi: 10.1002/cam4.4934 (PMC9844630; doi:10.1002/cam4.4934)
Supplement: Supplementary file 1 — Appendix S1 Supporting Information [file CAM4-12-379-s001.pdf]

Supplementary Information

Supplementary Table 1. Input variables for the Features II, PRS + Features II, Features III, and PRS + Features III models. Abbreviations: body mass index (BMI), C-reactive protein (CRP), glycated hemoglobin (HbA1c), insulin-like growth factor 1 (IGF-1) and polygenic risk score (PRS).

	**Machine Learning Model**			
**Input Variables**	*1) Features II*	*2) PRS + Features II*	*3) Features III*	*4) PRS + Features III*
**Genetic**		PRS		PRS
**Demographics**	Age	Age	Age	Age
	Father's history	Father's history	Father's history	Father's history
	Sibling history	Sibling history	Sibling history	Sibling history
	Ethnicity	Ethnicity	Ethnicity	Ethnicity
**Clinical Measurements**	BMI	BMI	BMI	BMI
**Laboratory values**	HbA1c	HbA1c	HbA1c	HbA1c
	CRP	CRP	CRP	CRP
	IGF-1	IGF-1	IGF-1	IGF-1
**Medical History**	Smoking status	Smoking status	Smoking status	Smoking status
			Number of sex partners	Number of sex partners
			Diabetes diagnosis	Diabetes diagnosis
			Diabetes medication	Diabetes medication

Supplementary Table 2. Performance metrics of the Features II, PRS + Features II, Features III, and PRS + Features III models. Abbreviations: Area under the receiver operating characteristic (AUROC); diagnostic odds ratio (DOR); likelihood ratio positive (LR+), likelihood ratio negative (LR-), polygenic risk score (PRS).

	Features II only	*PRS + Features II*	*Features III only*	*PRS + Features III*
**AUROC**	0.742<br>(0.708, 0.774)	0.788<br>(0.758, 0.819)	0.742<br>(0.708, 0.774)	0.788<br>(0.758, 0.819)
**Sensitivity**	0.800<br>(0.736, 0.864)	0.800<br>(0.736, 0.864)	0.800<br>(0.736, 0.864)	0.800<br>(0.736, 0.864)
**Specificity**	0.560<br>(0.553, 0.567)	0.629<br>(0.622, 0.636)	0.560<br>(0.553, 0.567)	0.629<br>(0.622, 0.636)
**DOR**	5.095<br>(4.694, 5.496)	6.783<br>(6.382, 7.184)	5.095<br>(4.694, 5.496)	6.783<br>(6.382, 7.184)
**LR+**	1.819<br>(1.676, 1.974)	2.157<br>(1.986, 2.341)	1.819<br>(1.676, 1.974)	2.157<br>(1.986, 2.341)
**LR-**	0.357<br>(0.259, 0.492)	0.318<br>(0.231, 0.438)	0.357<br>(0.259, 0.492)	0.318<br>(0.231, 0.438)

Supplementary Figure 1. SHapely Additive ExPlanations plots of the (**A**) Features I, (**B**) PRS + Features I, (**C**) Features II, (**D**) PRS + Features II model, and (**E**) Minimal Features models.

(A)

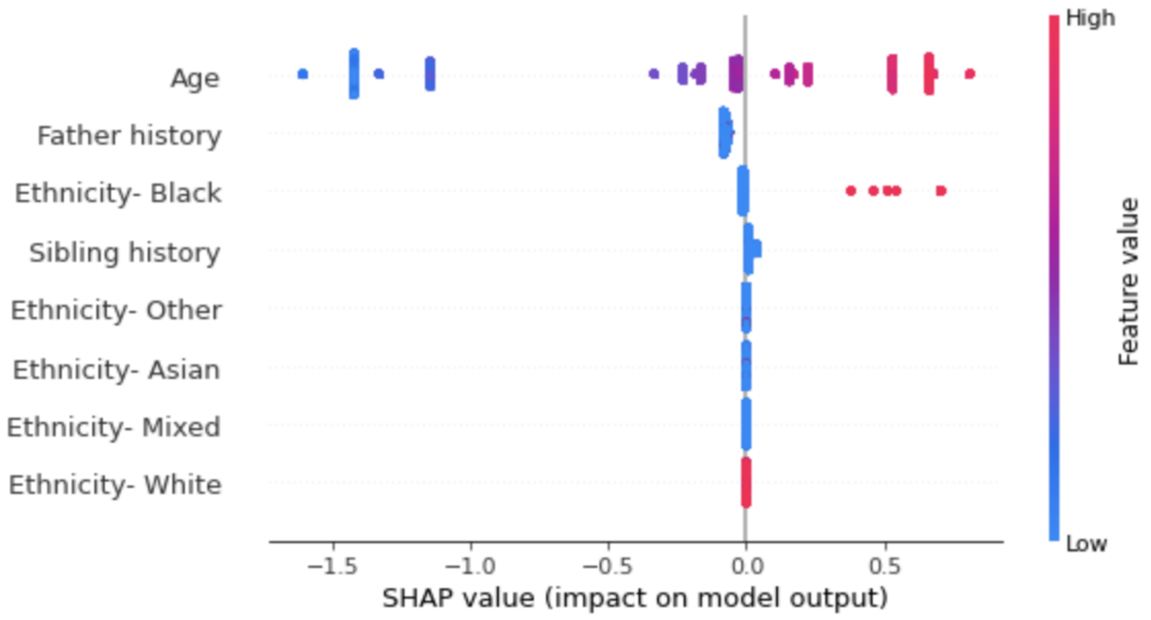

(B)

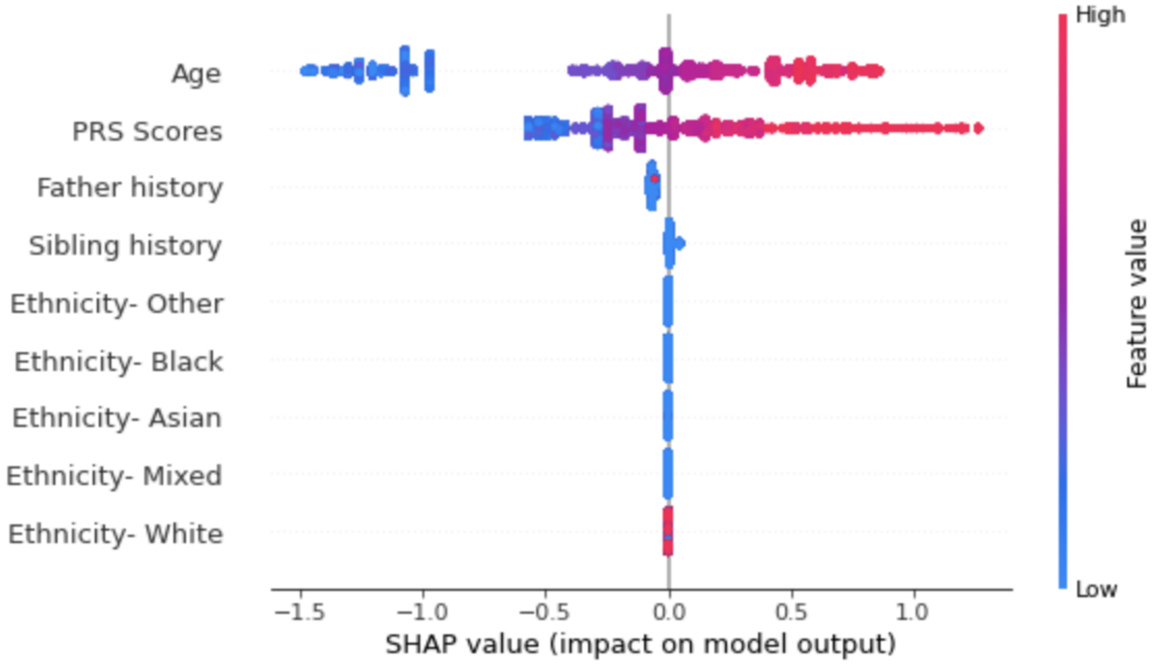

(C) Glycated hemoglobin in the Features II model was found to be a predictor of high importance, although combining the PRS data to this model substantially reduced its importance. The observed inverse relationship between HbA1c and PCa risk may be explained by the reduction of insulin growth factor and testosterone in diabetes, which can create a physiological environment that is unfavorable to PCa development.[1] Given that PCa in younger men has a strong genetic component,[2] the influence of diabetic control and PRS on PCa may be different in this age group.

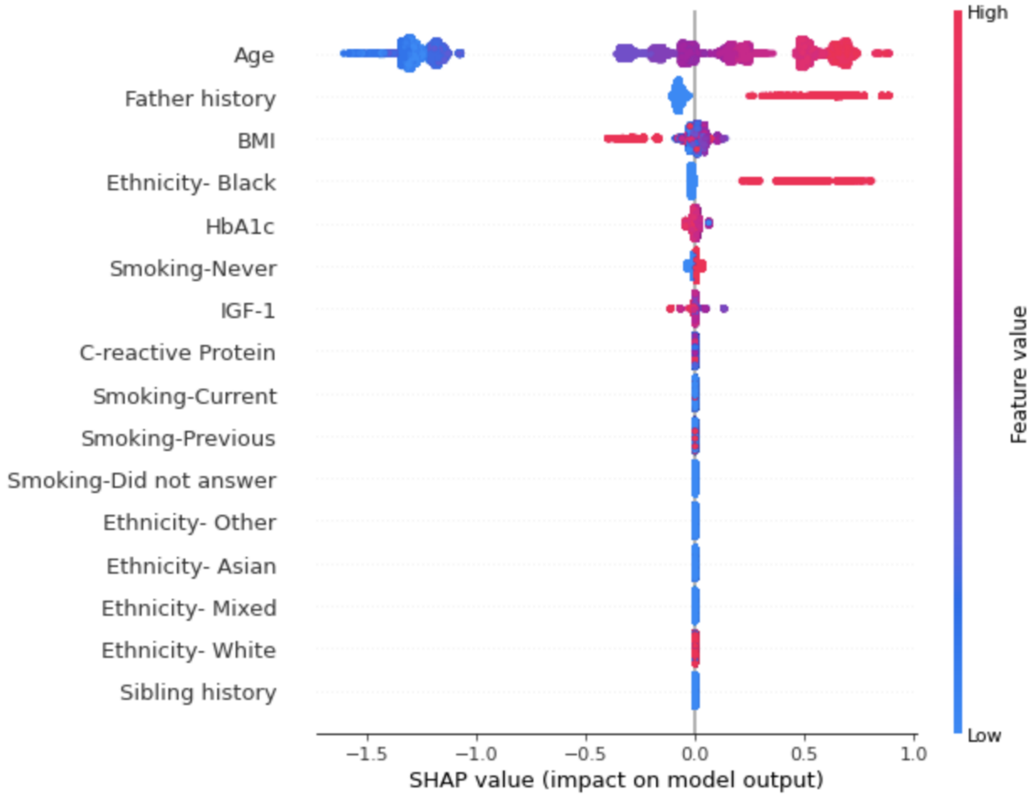

(D)

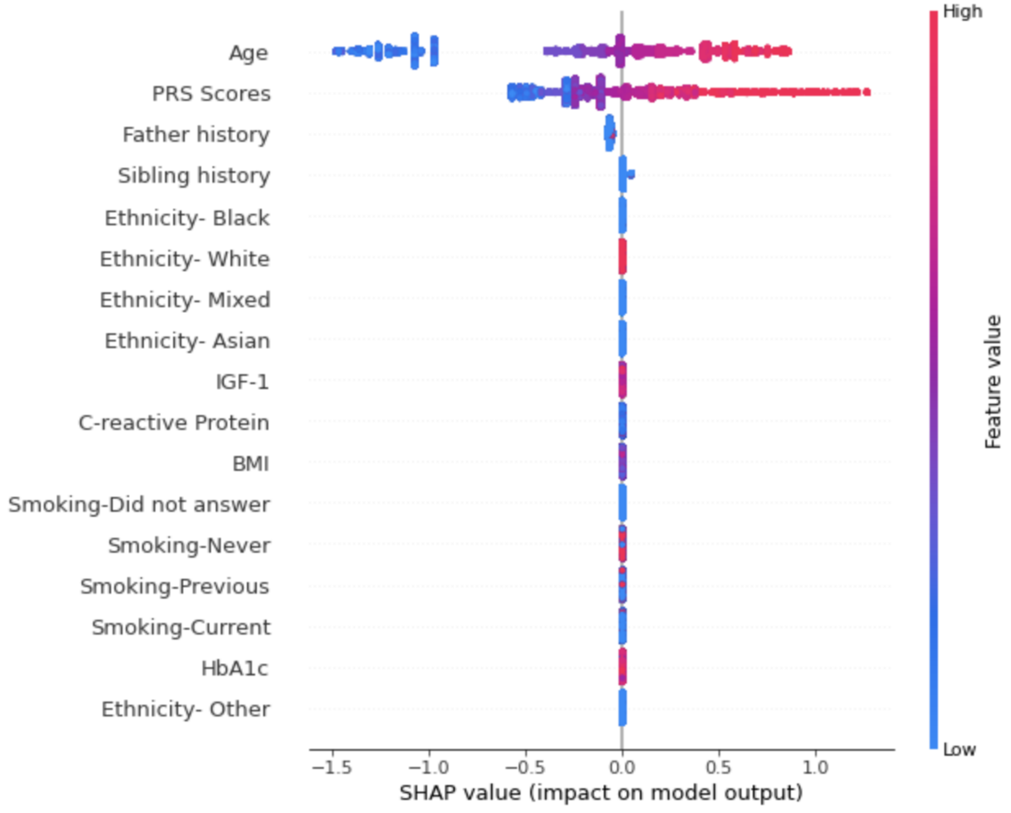

(E)

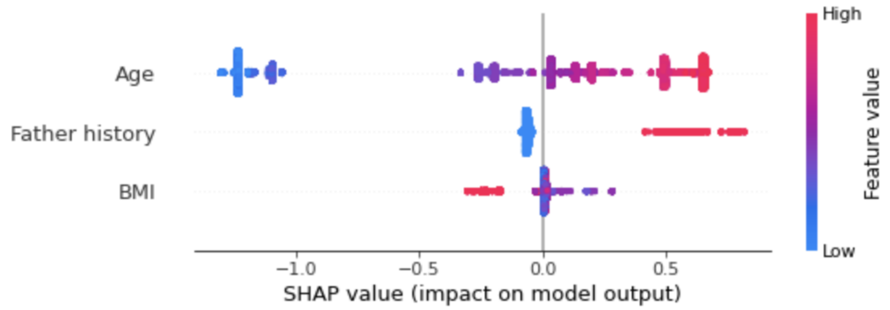

References

1. Kasper JS, Liu Y, Pollak MN, Rifai N, Giovannucci E. Hormonal profile of diabetic men and the potential link to prostate cancer. Cancer Causes Control. 2008 Sep 1;19(7):703–10.
2. Salinas CA, Tsodikov A, Ishak-Howard M, Cooney KA. Prostate Cancer in Young Men: An Important Clinical Entity. Nat Rev Urol. 2014 Jun;11(6):317–23.
